# Screening and Functional Prediction of Key Candidate Genes in Hepatitis B Virus-Associated Hepatocellular Carcinoma

**DOI:** 10.1155/2020/7653506

**Published:** 2020-10-09

**Authors:** Xia Chen, Ling Liao, Yuwei Li, Hengliu Huang, Qing Huang, Shaoli Deng

**Affiliations:** Department of Laboratory Medicine, Daping Hospital, Army Medical University (Third Military Medical University), Chongqing 400042, China

## Abstract

**Background:**

The molecular mechanism by which hepatitis B virus (HBV) induces hepatocellular carcinoma (HCC) is still unknown. The genomic expression profile and bioinformatics methods were used to investigate the potential pathogenesis and therapeutic targets for HBV-associated HCC (HBV-HCC).

**Methods:**

The microarray dataset GSE55092 was downloaded from the Gene Expression Omnibus (GEO) database. The data was analyzed by the bioinformatics software to find differentially expressed genes (DEGs). Gene Ontology (GO) enrichment analysis, Kyoto Encyclopedia of Genes and Genomes (KEGG) pathway analysis, ingenuity pathway analysis (IPA), and protein-protein interaction (PPI) network analysis were then performed on DEGs. The hub genes were identified using Centiscape2.2 and Molecular Complex Detection (MCODE) in the Cytoscape software (Cytoscape_v3.7.2). The survival data of these hub genes was downloaded from the Gene Expression Profiling Interactive Analysis (GEPIA).

**Results:**

A total of 2264 mRNA transcripts were differentially expressed, including 764 upregulated and 1500 downregulated in tumor tissues. GO analysis revealed that these DEGs were related to the small-molecule metabolic process, xenobiotic metabolic process, and cellular nitrogen compound metabolic process. KEGG pathway analysis revealed that metabolic pathways, complement and coagulation cascades, and chemical carcinogenesis were involved. Diseases and biofunctions showed that DEGs were mainly associated with the following diseases or biological function abnormalities: cancer, organismal injury and abnormalities, gastrointestinal disease, and hepatic system disease. The top 10 upstream regulators were predicted to be activated or inhibited by *Z*-score and identified 25 networks. The 10 genes with the highest degree of connectivity were defined as the hub genes. Cox regression revealed that all the 10 genes (CDC20, BUB1B, KIF11, TTK, EZH2, ZWINT, NDC80, TPX2, MELK, and KIF20A) were related to the overall survival.

**Conclusion:**

Our study provided a registry of genes that play important roles in regulating the development of HBV-HCC, assisting us in understanding the molecular mechanisms that underlie the carcinogenesis and progression of HCC.

## 1. Introduction

Hepatocellular carcinoma (HCC) is a highly malignant disease with high morbidity and mortality worldwide and is one of the leading causes of tumor mortality in the world [1–3]. HCC represents the result of a complex and heterogeneous malignant process that occurs in the context of an underlying progressive liver dysfunction [4]. HCC often arises from genetic mutations that alter the metabolic pathways, which therefore induces a disordered cell proliferation [4]. The majority of the HCC cases (approximately 80%) are associated with chronic hepatitis B virus (HBV) or hepatitis C virus (HCV) infections [5]. In China, chronic hepatitis caused by HBV is considered as the most important cause for the occurrence and development of liver cancer [6]. Although there are several reports on the pathogenesis of liver cancer caused by HBV, it still requires further elucidation. At present, the treatment of liver cancer mainly involves surgical treatment, but the 5-year survival rate associated with it remained low [7]. The main reasons for the limited effect of surgical treatment include low early diagnostic rate and high postoperative recurrence rate, and there is still a lack of effective adjuvant therapy beyond surgery. In recent years, the underlying molecular mechanisms of HCC pathogenesis were better understood. The development of HCC is a complex, multistep process that is associated with sustained inflammatory damage, including hepatocellular necrosis and regeneration, with fibrotic deposition [8]. However, there is currently a lack of overall understanding with regard to the pathogenesis of HCC in terms of pathways and network crosstalk. Therefore, an in-depth study of the molecular mechanisms and therapeutic targets in the occurrence and development of liver cancer has become a hot and key research topic.

The Gene Expression Omnibus (GEO, http://www.ncbi.nlm.nih.gov/geo/) is an international public storage database that includes data based on high-throughput chip and second-generation sequencing functional genome datasets uploaded by the research community [9]. Ingenuity pathway analysis (IPA) (http://www.ingenuity.com) is an integrated software application based on cloud computing and can be used to analyze the genome, miRNA, single-nucleotide polymorphism (SNP), chips, metabolism, protein, and RNA-Seq experiment and various small-scale experiment data, to build the model of the interaction [10]. It not only screen the molecular disease information rapidly in the early stage of the study, providing a scientific basis for the research design, but also carry out an in-depth data mining in the later stage of the study to build a complete biological system of molecular data comprehensively [10].

In this study, the original gene chip expression profile dataset GSE55092 from the GEO database containing a total of 140 samples, including 91 normal liver tissue samples and 49 HBV-HCC tissue samples, was analyzed. The obtained differentially expressed genes (DEGs) were analyzed using the Database for Annotation, Visualization and Integration Discovery (DAVID, https://david.ncifcrf.gov/) database for Gene Ontology (GO) functional annotation and Kyoto Encyclopedia of Genes and Genomes (KEGG) pathway analysis. The canonical pathways, diseases and biofunctions, upstream regulator analysis, regulator effects, and networks of DEGs were analyzed using QIAGEN's IPA analysis software. The protein-protein interaction (PPI) networks were constructed from the DEGs using the search tool for retrieving interacting genes/proteins (STRING) database. The key genes were then identified, and the corresponding modules were constructed according to the PPI network. Finally, the Gene Expression Profiling Interactive Analysis (GEPIA) was used to analyze the relationships between the hub genes and patient prognosis. Therefore, this study is aimed at performing an integrative analysis of all available high-throughput gene expression data on HBV-HCC in patients to elucidate the key genes involved in the molecular pathogenesis of it.

## 2. Materials and Methods

### 2.1. Data Source

The raw data of this study is obtained from the GEO database of the National Center for Biotechnology Information (NCBI), and the NCBI-GEO is an open microarray and a next-generation sequencing database. The accession number GSE55092 was based on GPL570 (Affymetrix Human Genome U133 Plus 2.0 Array) [11].

### 2.2. Data Preprocessing and Differential Expression Analysis

The data processing was assisted by Beijing Kangshengsaike Technology Co., LTD (Beijing, China). After the original data was normalized by the Expression Console (EC), the Affymetrix® Transcriptome Analysis Console (TAC) software was used to analyze the gene differences among the samples. If *P* < 0.05, ∣log fold change (FC) | >2, then the gene was considered to be differentially expressed.

### 2.3. GO Enrichment Analysis and KEGG Pathway Enrichment Analysis

The GO enrichment analysis and KEGG pathway enrichment analysis were performed using the DAVID database [12]. *P* < 0.05 was considered to be statistically significant. Also, the top 20 GO terms and the KEGG pathways were selected.

### 2.4. Ingenuity Pathway Analysis

To carry out an in-depth biological information analysis of 2264 common differential genes, canonical pathways, diseases and biofunctions, upstream regulator analysis, regulator effects, and networks of 2264 common differential genes were analyzed by QIAGEN's IPA analysis software. Fisher's exact test and Benjamini-Hochberg correction were used to identify significantly enriched DEGs as members of pathways and functional categories [13]. Upstream analysis was conducted based on the interaction relationship between transcriptional regulators (TR) and their target genes in Ingenuity Knowledge Base [10]. It was predicted by overlap *P* value and activation *Z*-score, in which the overlap *P* value was calculated based on the intersection of regulatory objects and differential genes in datasets reported in the literature, and *P* < 0.05 was considered to be significant. The *Z*-score was calculated based on the expression association between regulators and genes, and weighted correction was done according to the interaction type and data deviation. *Z*‐score > 2 or <-2 was considered as significant. The underlying network of our algorithms was based on the Ingenuity Knowledge Base [10]. The score of the networks was calculated based on the *P* value, reflecting the probability that the molecules of the dataset appear in the network as a random process. The score was obtained by the -log of right-tailed Fisher's exact test.

### 2.5. PPI Network Construction and Analysis of Modules

The PPI of common DEGs was analyzed using a STRING online database (http://string-db.org), and the common DEGs of PPI network visualization have been realized by using the Cytoscape software (http://www.cytoscape.org/) [14]. The search clustered subnetworks were used using Cytoscape MCODE. The default parameters were as follows: degree cutoff ≥ 2, node score cutoff ≥ 0.2, *K*‐core ≥ 2, and max depth = 100. By calculating the centrality parameters of each node, CentiScaPe was used for finding the most important nodes in a network.

### 2.6. Validation and Survival Analysis of Key Hub Genes

The protein expression and raw survival data were downloaded from the GEPIA website (http://gepia.cancer-pku.cn/). Cancer type was restricted by liver hepatocellular carcinoma (LIHC), and the expressions of CDC20, BUB1B, KIF11, TTK, EZH2, ZWINT, NDC80, TPX2, MELK, and KIF20A were obtained.

## 3. Results

### 3.1. Differentially Expressed mRNAs in HBV-HCC

**Figure 1:**
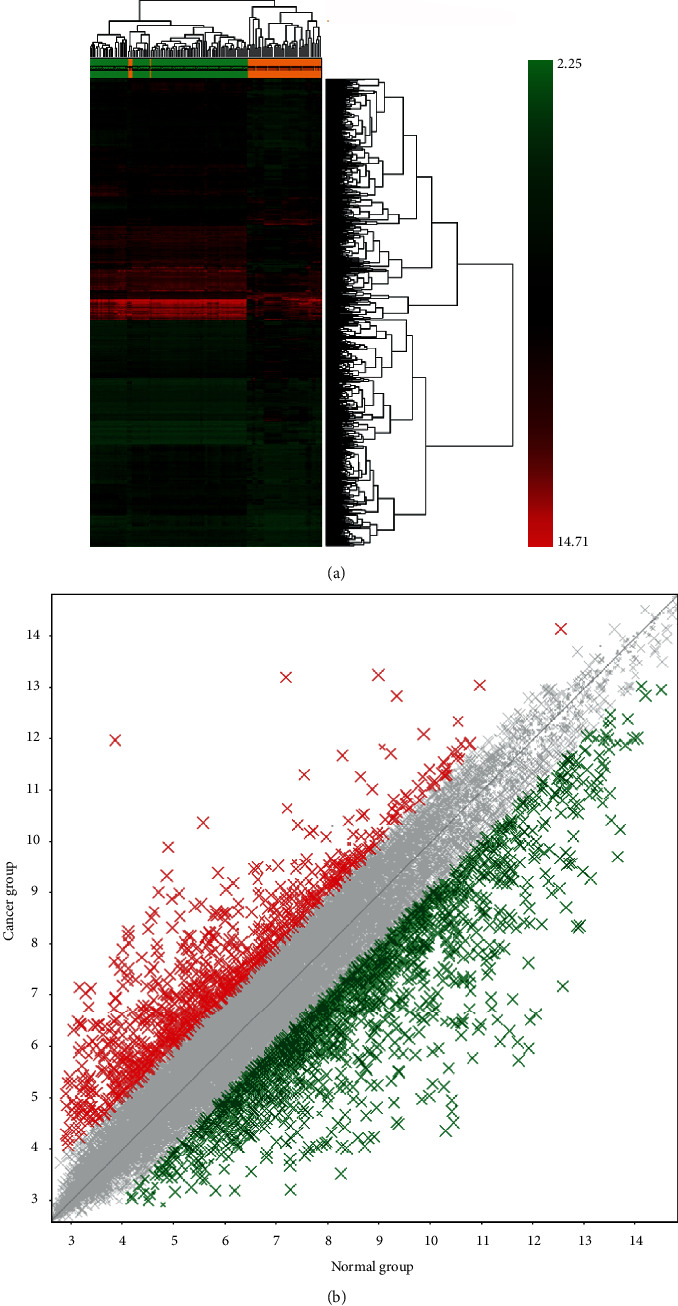
Identification of aberrantly expressed mRNAs. (a) Heat map of differentially expressed mRNA. (b) The scatter plot of differentially expressed mRNA. Red color represents upregulation of differential genes, while green color represents downregulation of differential genes. *P* < 0.05 and ∣logFC | >2 were chosen as the cutoff criteria.

The NCBI-GEO database is an open database, and the clinicopathological characteristics of the patients with HBV-associated HCC are shown in Table 1[11]. According to the filtering criteria of *P* < 0.05 and ∣logFC | >2, a total of 2264 differentially expressed mRNAs were identified (Table S1). Among these, 764 mRNAs were upregulated, and 1500 mRNAs were downregulated. As shown in Figure 1(a), the hierarchical clustering analysis was performed for these 2264 aberrantly expressed mRNAs. The results showed that the expression level of each transcript was represented by a color, ranging from green (low) to red (high). Each column and each row represents one group and one mRNA, respectively. The scatter plot in Figure 1(b) showed the number of DEGs identified from each dataset.

### 3.2. GO and KEGG Pathway Enrichment Analysis

**Figure 2:**
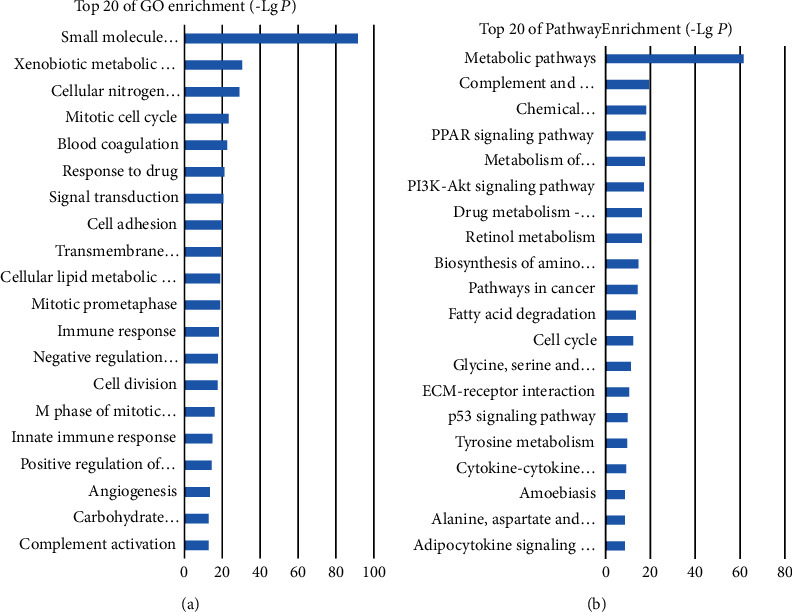
Functional annotation of DEGs by DAVID. (a) The top 20 GO terms related to mRNA dysregulation. (b) The top 20 of KEGG pathway of DEGs in HBV-HCC. The value of -Lg*P* indicates the significance of the GO and KEGG signaling pathway. Differences were considered statistically significant at *P* < 0.05.

GO analysis revealed the associated functions of these abnormally expressed mRNAs. A total of 712 GO terms have been shown to be significantly enriched. The most highly enriched GO terms of dysregulated mRNAs were associated with the small-molecule metabolic process (GO:0044281), xenobiotic metabolic process (GO:0006805), and cellular nitrogen compound metabolic process (GO:0034641) (Figure 2(a)). KEGG showed that the genes were mainly enriched in metabolic pathways, complement and coagulation cascades, and chemical carcinogenesis (Figure 2(b)). Meanwhile, GO and KEGG analyses were performed for upregulated and downregulated DEGs, respectively, and the results are shown in Figure S1. Moreover, as shown in Figure S2, the IPA results of canonical pathways showed that the DEGs were enriched in different pathways, among which LXR/RXR activation, FXR/RXR activation, and LPS/IL-1-mediated inhibition of RXR functions are the most significantly affected.

### 3.3. Diseases and Biofunctions

**Figure 3:**
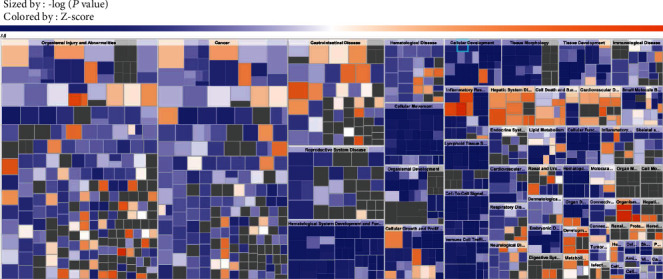
Diseases and biofunctions. In the hierarchical clustering of heat map, each individual colored rectangle is a particular biological function or disease. The patch size is determined by *P* value. The smaller the *P* value, the larger the patch is. The plaque color is determined by *Z*-score; the *Z*‐score > 2 and <-2 is considered meaningful. Blue color indicates suppressed disease or biological function, and orange indicates that the disease or biological function is activated. Grey indicates that the *Z*-score for the biological function or disease is unknown.

IPA can be used to study the potential link between biological function and disease. The differences between the genomes of HCC and other diseases were compared to detect the possible correlations between HCC and other diseases at the genome level. As shown in Figure 3, through the application of disease and biological function enrichment analysis, it was revealed that among the 2264 genes with common differences, the genes were mainly related to the following diseases or biological function abnormalities: cancer, organismal injury and abnormalities, gastrointestinal disease, and hepatic system disease (Table S2). The diseases and biofunctions were also presented as a histogram (Figure S3).

### 3.4. Upstream Regulator Analysis

**Figure 4:**
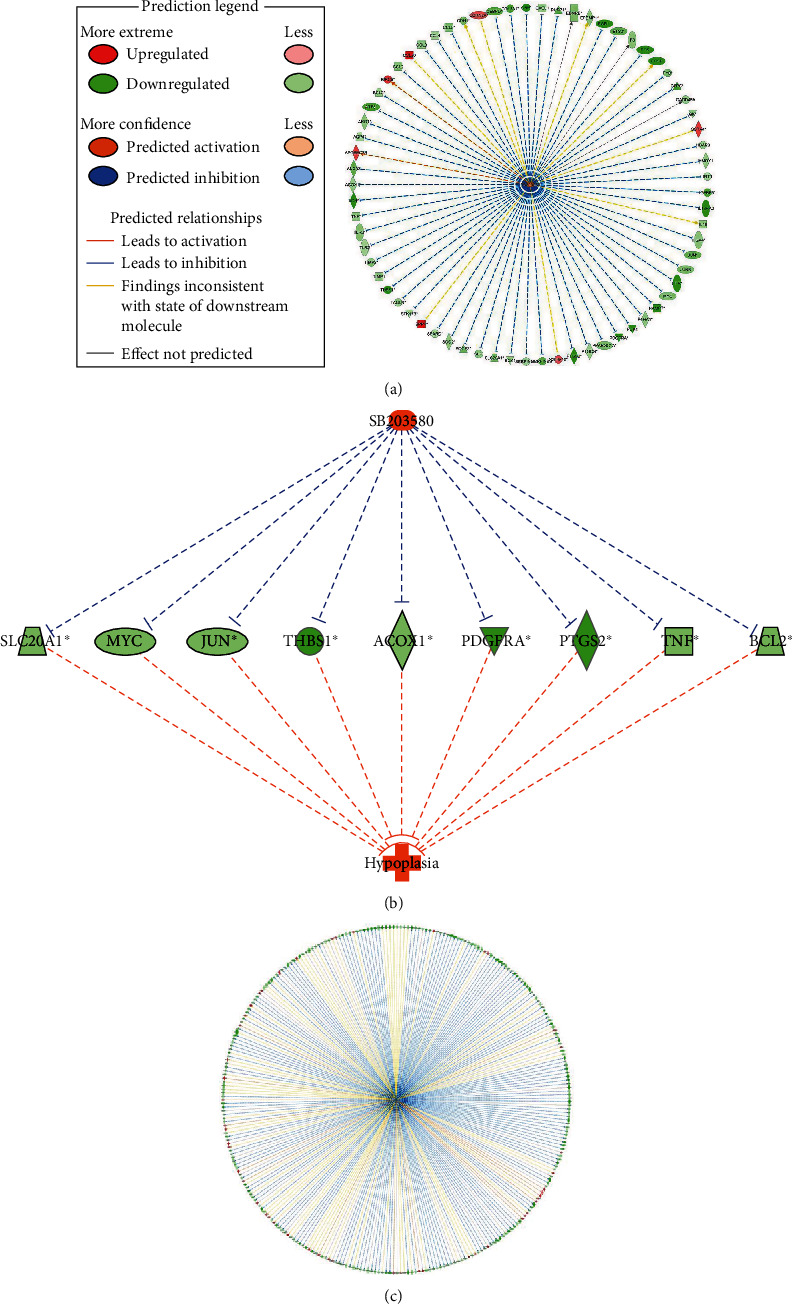
Upstream regulator analysis of differentially expressed genes in HBV-HCC. (a) The relevant network of SB203580. (b) SB203580 regulates network molecules related to hypoplasia. (c) The relevant network of lipopolysaccharide.

The top 10 upstream regulators predicted to be activated by *Z*-score are presented in Table 2. SB203580 was the most predicted activated upstream regulators by *Z*-score for the dataset involving HBV-HCC (Figure 4(a)). At the same time, SB203580 regulated some downstream genes related to hypoplasia (Figure 4(b)), including ACOX1, BCL2, JUN, MYC, PDGFRA, PTGS2, SLC20A1, THBS1, and TNF. The top 10 upstream regulators that were predicted to be inhibited by *Z*-score are presented in Table 3. The most predicted inhibited upstream regulators by *Z*-score included lipopolysaccharide (Figure 4(c)).

### 3.5. Network Analysis

**Figure 5:**
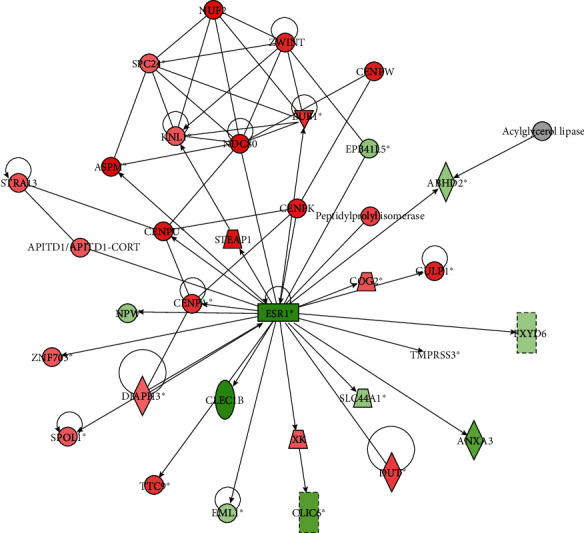
Top network identified by ingenuity pathway analysis. The top network is called “Cell Cycle, Cellular Assembly and Organization, DNA Replication, Recombination, and Repair”. Red represents the upregulation of genes, and green represents the downregulation of genes.

According to the input dataset, 25 networks were identified and listed in the decreasing order of significance (Table S3). The identified top network of genes unified the functional terms “Cell Cycle, Cellular Assembly and Organization, DNA Replication, Recombination, and Repair” (Figure 5). For the network with 33 focus molecules, ESR1 was found to be the central node.

### 3.6. Key Candidate Gene Identification with DEG PPI Network

**Figure 6:**
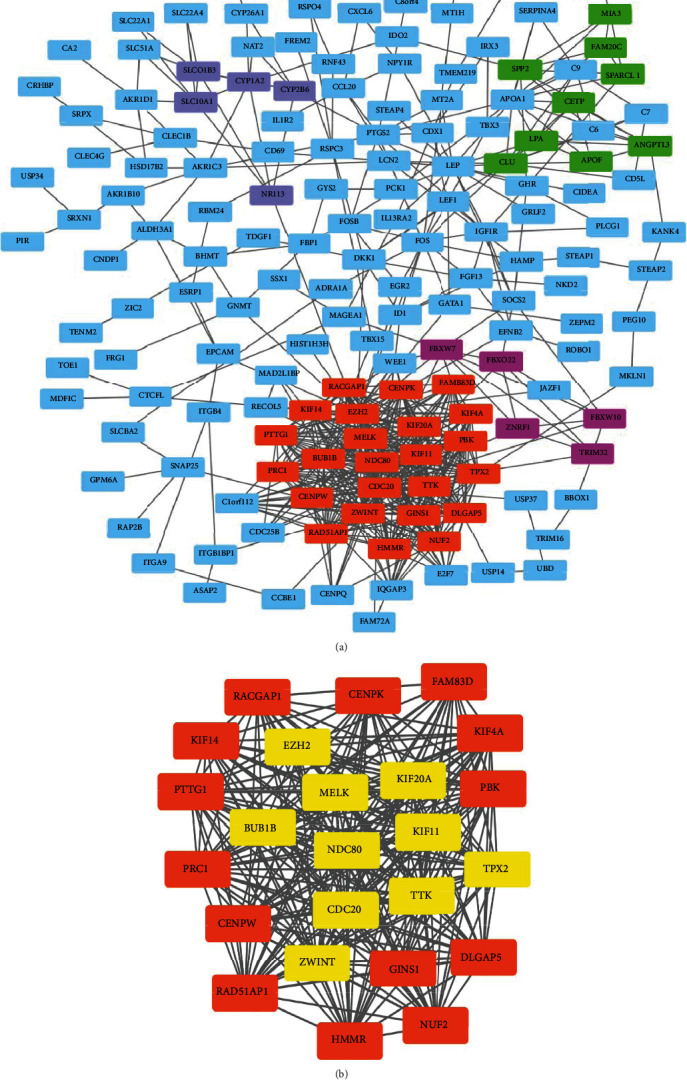
PPI network and module analysis. (a) PPI network of the most significant DEGs identified from GSE55092 was constructed. Four subnetworks were identified by Cytoscape MCODE. Genes in different subnetworks are shown in different colors, and blue nodes indicate other genes. (b) One most important module subnetwork was identified by Cytoscape MCODE. Yellow represents the hub genes.

The PPI network of DEGs was constructed by using the STRING online database and Cytoscape (Figure 6), and the top ten highly connected genes included CDC20, BUB1B, KIF11, TTK, EZH2, ZWINT, NDC80, TPX2, MELK, and KIF20A (Table 4). MCODE was used for module analysis of the PPI network, and the most significant was chosen for further pathway analyses based on the degree of importance.  Figure 6(b) shows the most important module in which all hub genes are contained.

### 3.7. Validation and Survival Analysis of Key Hub Genes

**Figure 7:**
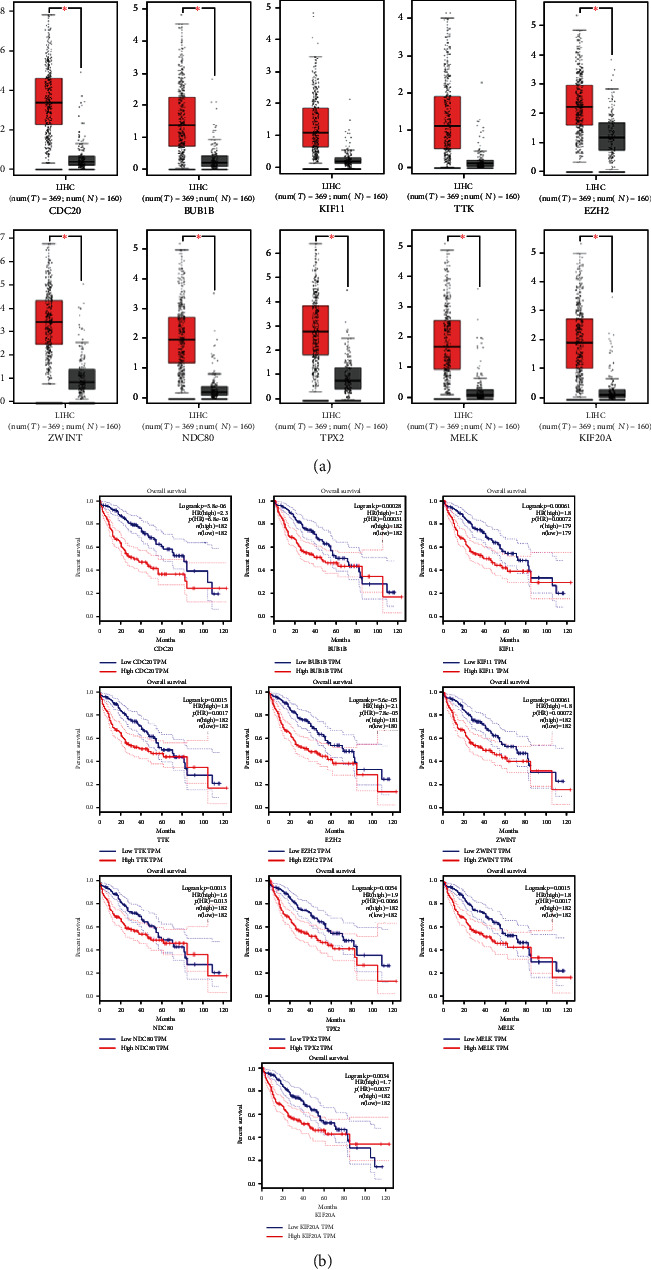
Validation of hub genes in the TCGA dataset. (a) CDC20, BUB1B, KIF11, TTK, EZH2, ZWINT, NDC80, TPX2, MELK, and KIF20A expressions in 369 LIHC patients compared with 160 normal samples. (b) Overall survival curves of CDC20, BUB1B, KIF11, TTK, EZH2, ZWINT, NDC80, TPX2, MELK, and KIF20A.

The key hub genes were validated in the TCGA dataset. All of the hub genes were upregulated in 369 LIHC samples as compared to 160 normal samples (Figure 7(a)). Among the 10 hub genes, all genes that showed association with the prognosis of HCC patients were found: CDC20 (*P* = 3.8*e* − 06), BUB1B (*P* = 0.0028), KIF11 (*P* = 0.00061), TTK (*P* = 0.0015), EZH2 (*P* = 5.6*e* − 05), ZWINT (*P* = 0.00061), NDC80 (*P* = 0.013), TPX2 (*P* = 0.00054), MELK (*P* = 0.0015), and KIF20A (*P* = 0.0034), respectively. The Kaplan-Meier analysis results are presented in Figure 7(b).

## 4. Discussion

In recent years, many studies have shown the involvement of HBV in the carcinogenesis, invasion, and metastasis of liver cells and also play a key role in the occurrence and development of liver cancer [15]. In the treatment of HCC, there is a lack of effective therapeutic target clinically [16]. The poor prognosis of HCC patients after treatment is mainly due to the high incidence of intrahepatic and extrahepatic metastasis of HCC [17]. However, the metastasis of cancer cells is a complicated process, and both intracellular and tumor microenvironmental factors can affect the metastatic ability of tumor cells. Most of the molecular mechanisms that mediate metastasis are still unclear. Therefore, further studies should be conducted to understand the molecular mechanisms of HBV-HCC development, invasion, and metastasis, which not only facilitates the early diagnosis of HCC but also assists in finding better drug targets for clinical treatment of HCC.

Based on microarray analysis, 2264 common DEGs were identified by studying the gene expression profiles. The GO function analysis of 2264 differential genes showed that the most highly enriched GO terms of HBV-HCC differential genes were included in the small-molecule metabolic process, xenobiotic metabolic process, and cellular nitrogen compound metabolic process. KEGG analysis showed that the genes were mainly enriched in metabolic pathways, complement and coagulation cascades, and chemical carcinogenesis. IPA revealed that the top significantly changed canonical pathways are related to FXR/RXR activation, LXR/RXR activation, and LPS/IL-1-mediated inhibition of RXR function, and this was consistent with that of the previous reports in the literature. We found that FXR/RXR and LXR/RXR are involved in many diseases, such as hypothalamic dysfunction [18], germ cell development [19], adipose stem cells (ASCs) [20], colorectal cancer (CRC) [21], and major depressive disorder [22]. A study has found that the stimulation of glutamine to ASCT2 expression partly involves the binding of FXR/RXR to the ASCT2 promoter [23], which might be the key to the proliferation and survival of HepG2 cells. LXR-mediated transactivation is coactivated by PGC-1*α* (peroxisome-proliferator-activated receptor-*γ*co-activator-1*α*), which in turn can restore the SREBP-1 isoform expression in HepG2 cells [24]. Therefore, this study helps us to elucidate the mechanisms of proliferation and invasion of HBV-HCC and to predict the progression of cancer.

According to the disease and biofunction network analysis, major diseases of HCC targets were screened. IPA revealed cancer, organismal injury and abnormalities, gastrointestinal disease, and hepatic system disease as the top diseases and biofunctions associated with these mRNAs. The goal of upstream regulator analysis is to identify molecules that are upstream of these genes in the dataset, and this might explain the observed changes in the expression. Also, the top 10 activated and inhibited regulators of various categories were selected, respectively. For instance, SB203580 inhibits proliferation and invasion of HepG2 cells by blocking the formation of oncogenic pSmad3L and smad2/3/4 complexes [25]. Lipopolysaccharide stimulates the activation of hepatic stellate cells through the TLR4 pathway and promotes angiogenesis in mouse HCC model [26]. A total of 25 networks based on input dataset were identified. The most important of these involves the biological importance of our data, which is related to the cell cycle, cellular assembly and organization, DNA replication, recombination, and repair. The central node is ESR1, which has been the focus of breast cancer research for some time and also has clinical implications in endometrial [27], ovarian [28], and other types of cancers.

PPI networks were constructed, and the following 10 hub genes were identified: CDC20, BUB1B, KIF11, TTK, EZH2, ZWINT, NDC80, TPX2, MELK, and KIF20A. The GEPIA was used to analyze the prognosis of these 10 hub genes, and the results showed that the expression levels of these 10 genes were associated with the prognosis of HCC patients. Abnormal expression of CDC20 appears in most of the human cancers [29]. Inhibition of CDC20 expression in HCC reduced cell proliferation and induced G2/M cell cycle arrest, showing a positive correlation with TNM staging [30]. Consistent with our research findings, increased expression of BUB1B is associated with poor prognosis in HCC patients [31]. KIF11 is highly expressed in blast crisis chronic myelogenous leukemia [32] and pancreatic cancer [33]. Previous reports have suggested that TTK promotes cell proliferation and invasion, and its functions include promoting the formation of mitotic checkpoint complexes, regulating cell division, responding to DNA damage, and promoting chromosome alignment [34]. Sudo et al. [35] have reported that EZH2 was significantly upregulated in HCC tissues when compared to those with corresponding nontumor specimens. ZWINT protein is shown to be elevated in HCC tissues and is associated with tumor size and number. The HCC patients with high ZWINT expression are associated with a high tumor recurrence rate. NDC80 participates in the pathogenesis of HCC through its proliferation and antiapoptotic effects and might be considered as a new target for HCC gene therapy [36]. Downregulation of TPX2 in human HCC can inhibit PI3K/AKT signal transduction, inhibit cell proliferation, and promote cell apoptosis [37]. MELK overexpression has been detected in a variety of human tumors, suggesting it as an important factor in tumorigenesis [38]. KIF20A is a member of the drive protein superfamily, which is mainly involved in the cellular mitotic process. KIF20A expression is significantly increased in HCC [39]. The top 10 hub genes were obtained by the PPI network and are closely related to tumor development and tumor progression, suggesting that these hub genes might act as prognostic markers and therapeutic targets in HCC.

In summary, our research identified several key candidate genes that are involved in HBV-HCC progression through an integrated bioinformatics analysis, which further contributes to the search of biomarkers and therapeutic targets for HBV-HCC. However, more molecular biology experiments are warranted to further explore the underlying mechanisms of these key candidate genes in the development of HBV-HCC.

## Figures and Tables

**Table 1:** Baseline characteristics of the patients with HBV-associated HCC.

Characteristic	
Male to female	10 : 1
Age (years)	57.7 ± 7.7
Total bilirubin (mg/dL)	0.88 ± 0.47
Albumin (g/dL)	3.91 ± 0.57
Alanine aminotransferase (U/L)	36.18 ± 17.8
Aspartate aminotransferase (U/L)	39.09 ± 17.0
*γ*-Glutamyltransferase (U/L)	93.9 ± 83.56
Tumor grade^a^	
G2 (%)	64
G3 (%)	27
G4 (%)	9
HBsAg-positive (%)	100
HBeAg-positive (%)	0
Anti-HBc (%)	100
Anti-HBe (%)	100

^a^Tumors were graded using the Edmondson-Steiner criteria. Plus-minus values are means ± SD.

**Table 2:** Top 10 upstream regulators that are predicted to be activated by *Z*-score.

Upstream regulator	Molecule type	*Z*-score	*P* value
*SB203580*	Chemical-kinase inhibitor	5.805	4.57*E* − 16
*RABL6*	Other	5.112	9.62*E* − 19
*U0126*	Chemical-kinase inhibitor	4.928	2.49*E* − 23
*Bisindolylmaleimide I*	Chemical-kinase inhibitor	4.246	0.000000677
*Pyrrolidine dithiocarbamate*	Chemical reagent	4.182	1.49*E* − 08
*Actinomycin D*	Chemical drug	4.156	1.97*E* − 13
*COL18A1*	Other	4.104	3.59*E* − 10
*SP600125*	Chemical-kinase inhibitor	3.948	3.16*E* − 12
*SB202190*	Chemical-kinase inhibitor	3.848	2.14*E* − 08
*NR0B2*	Ligand-dependent nuclear receptor	3.666	0.00000586

**Table 3:** Top 10 upstream regulators that are predicted to be inhibited by *Z*-score.

Upstream regulator	Molecule type	*Z*-score	*P* value
*Lipopolysaccharide*	Chemical drug	-6.951	3.4*E* − 59
*Poly rI:rC-RNA*	Biologic drug	-6.267	4.08*E* − 18
*IL1B*	Cytokine	-6.089	1.75*E* − 44
*IFNG*	Cytokine	-5.785	2.93*E* − 35
*TNF*	Cytokine	-5.607	5.37*E* − 60
*TLR3*	Transmembrane receptor	-5.41	7.11*E* − 11
*CREB1*	Transcription regulator	-5.368	4.87*E* − 22
*PDGF BB*	Complex	-5.235	2.66*E* − 30
*HNF4A*	Transcription regulator	-5.153	1.28*E* − 17
*TP53*	Transcription regulator	-5.14	2.72*E* − 49

**Table 4:** The degree values of top 10 hub genes.

Gene symbol	Gene description	Fold change	Degree
*CDC20*	Cell division cycle 20	4.16	36
*BUB1B*	BUB1 mitotic checkpoint serine	6.17	30
*KIF11*	Kinesin family member 11	3.66	29
*TTK*	TTK protein kinase	5.02	28
*EZH2*	Enhancer of zeste homolog 2	3.24	28
*ZWINT*	ZW10 interacting kinetochore protein	3.55	27
*NDC80*	NDC80 kinetochore complex component	7.72	27
*TPX2*	Microtubule nucleation factor	2.31	27
*MELK*	Maternal embryonic leucine zipper kinase	7.15	27
*KIF20A*	Kinesin family member 20A	5.58	26

## Data Availability

The data used to support the findings of this study are available from the corresponding author upon reasonable request.

## Supplementary Materials

Supplementary Materials Figure S1: GO function annotation and KEGG pathway analysis of differentially expressed genes (DEGs) by DAVID. Figure S2: the most representative canonical pathways associated with HBV-HCC are shown from IPA. Figure S3: diseases and biofunctions are presented in the form of histogram. Table S1: raw data of key differentially expressed genes in HBV-HCC. Table S2: the detailed data about diseases and biofunctions. Table S3: IPA predicted networks that are associated with HBV-HCC.
