# Supplementary material for: Screening and Functional Prediction of Key Candidate Genes in Hepatitis B Virus-Associated Hepatocellular Carcinoma
Source: Biomed Res Int. 2020 Oct 9;2020:7653506. doi: 10.1155/2020/7653506 (PMC7568806; doi:10.1155/2020/7653506)
Supplement: Supplementary Materials — Figure S1: GO function annotation and KEGG pathway analysis of differentially expressed genes (DEGs) by DAVID. Figure S2: the most representative canonical pathways associated with HBV-HCC are shown from IPA. Figure S3: diseases and biofunctions are presented in the form of histogram. Table S1: raw data of key differentially expressed genes in HBV-HCC. Table S2: the detailed data about diseases and biofunctions. Table S3: IPA predicted networks that are associated with HBV-HCC. [file 7653506.f1.zip › Supplemental Table 3.docx]

| ID | Score | Focus Molecules | Top Diseases and Functions |
| --- | --- | --- | --- |
| 1 | 40 | 33 | Cell Cycle, Cellular Assembly and Organization, DNA Replication, Recombination, and Repair |
| 2 | 36 | 31 | Cell Morphology, Cellular Assembly and Organization, Cellular Function and Maintenance |
| 3 | 34 | 30 | Carbohydrate Metabolism, Neurological Disease, Developmental Disorder |
| 4 | 32 | 29 | Post-Translational Modification, Cell-To-Cell Signaling and Interaction, Drug Metabolism |
| 5 | 30 | 28 | Cell Signaling, Molecular Transport, Nucleic Acid Metabolism |
| 6 | 29 | 28 | Amino Acid Metabolism, Small Molecule Biochemistry, Cancer |
| 7 | 29 | 28 | Tissue Morphology, Cancer, Organismal Injury and Abnormalities |
| 8 | 29 | 28 | Lipid Metabolism, Small Molecule Biochemistry, Metabolic Disease |
| 9 | 29 | 28 | Cellular Compromise, DNA Replication, Recombination, and Repair, Metabolic Disease |
| 10 | 28 | 27 | Small Molecule Biochemistry, Vitamin and Mineral Metabolism, Developmental Disorder |
| 11 | 28 | 27 | Molecular Transport, Developmental Disorder, Drug Metabolism |
| 12 | 28 | 27 | Post-Translational Modification, Neurological Disease, Organismal Injury and Abnormalities |
| 13 | 28 | 27 | Energy Production, Lipid Metabolism, Small Molecule Biochemistry |
| 14 | 28 | 27 | Cell Cycle, Embryonic Development, Organismal Development |
| 15 | 27 | 27 | Connective Tissue Development and Function, Embryonic Development, Organ Development |
| 16 | 26 | 27 | Cardiovascular System Development and Function, Cell-To-Cell Signaling and Interaction, Cellular Movement |
| 17 | 26 | 26 | Cancer, Connective Tissue Disorders, Organismal Injury and Abnormalities |
| 18 | 26 | 26 | Visual System Development and Function, Embryonic Development, Organismal Development |
| 19 | 26 | 26 | Cellular Movement, Cancer, Organismal Injury and Abnormalities |
| 20 | 26 | 26 | Cellular Development, Cellular Growth and Proliferation, Cell-To-Cell Signaling and Interaction |
| 21 | 26 | 28 | Cancer, Organismal Injury and Abnormalities, Reproductive System Disease |
| 22 | 26 | 26 | Drug Metabolism, Small Molecule Biochemistry, Endocrine System Development and Function |
| 23 | 25 | 27 | Lipid Metabolism, Small Molecule Biochemistry, Organismal Injury and Abnormalities |
| 24 | 25 | 25 | Nervous System Development and Function, Tissue Development, Cell Death and Survival |
| 25 | 25 | 25 | Cell Morphology, Cellular Compromise, Cardiovascular Disease |
