# Supplementary figures and images for: Screening and Functional Prediction of Key Candidate Genes in Hepatitis B Virus-Associated Hepatocellular Carcinoma
Source: Biomed Res Int. 2020 Oct 9;2020:7653506. doi: 10.1155/2020/7653506 (PMC7568806; doi:10.1155/2020/7653506)

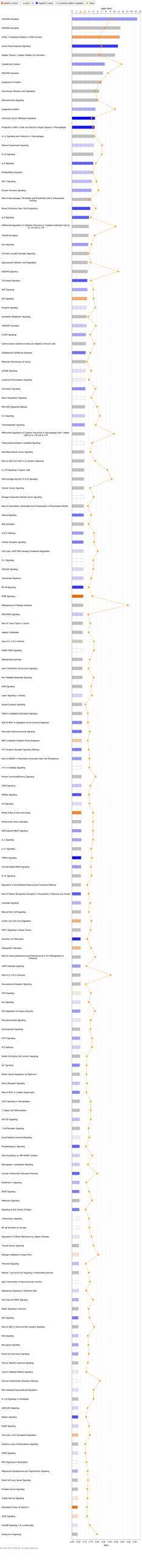

Supplement: Supplementary Materials — Figure S1: GO function annotation and KEGG pathway analysis of differentially expressed genes (DEGs) by DAVID. Figure S2: the most representative canonical pathways associated with HBV-HCC are shown from IPA. Figure S3: diseases and biofunctions are presented in the form of histogram. Table S1: raw data of key differentially expressed genes in HBV-HCC. Table S2: the detailed data about diseases and biofunctions. Table S3: IPA predicted networks that are associated with HBV-HCC. [file 7653506.f1.zip › Supplemental Figure 2.png]

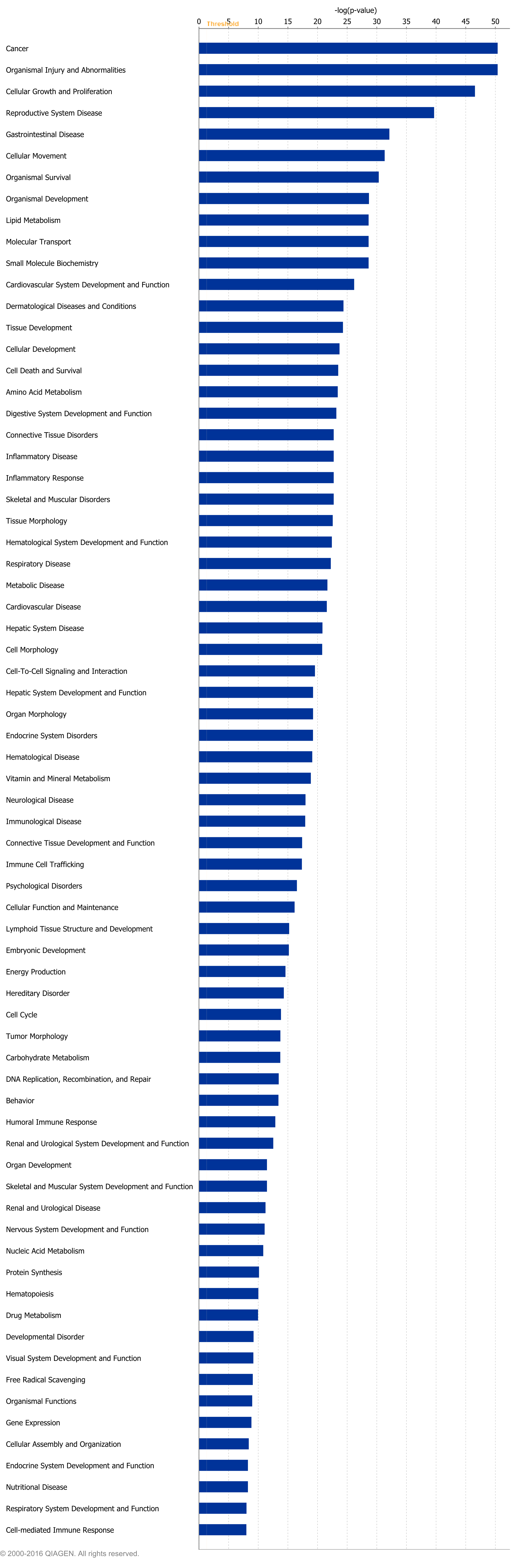

Supplement: Supplementary Materials — Figure S1: GO function annotation and KEGG pathway analysis of differentially expressed genes (DEGs) by DAVID. Figure S2: the most representative canonical pathways associated with HBV-HCC are shown from IPA. Figure S3: diseases and biofunctions are presented in the form of histogram. Table S1: raw data of key differentially expressed genes in HBV-HCC. Table S2: the detailed data about diseases and biofunctions. Table S3: IPA predicted networks that are associated with HBV-HCC. [file 7653506.f1.zip › Supplemental Figure 3.png]
